# Early anti-VEGF treatment for hemorrhagic occlusive retinal vasculitis as a complication of cataract surgery

**DOI:** 10.1186/s12886-017-0632-y

**Published:** 2017-12-06

**Authors:** Konstantinos Andreanos, Petros Petrou, George Kymionis, Dimitrios Papaconstantinou, Ilias Georgalas

**Affiliations:** 1 First Division of Ophthalmology, School of Medicine, National and Kapodistrian University of Athens, “G. Gennimatas” General Hospital of Athens, Athens, Greece; 2 22str Digeni E.O.K.A, Nea Penteli, 15236 Athens, Greece

**Keywords:** Vancomycin, Hemorrhagic occlusive retinal vasculitis, Cataract surgery, Anti-VEGF

## Abstract

**Background:**

We report a case of hemorrhagic occlusive retinal vasculitis (HORV) after prophylactic intracameral vancomycin use during an uneventful cataract surgery treated with early anti-VEGF treatment.

**Case presentation:**

A 51-year-old female underwent uneventful cataract surgery with prophylactic intracameral vancomycin during the procedure. On the seventh post-operative-day, she presented with sudden painful, visual loss. Fundus examination revealed peripheral hemorrhagic retinal vasculitis. She received anti-VEGF therapy to prevent further vision loss and retinal neovascularization due to extensive retinal ischemia. At the 6-month follow-up visit, visual acuity was 20/20 with no sign of neovascularization.

**Conclusions:**

Postoperative HORV is a devastating condition that can occur after otherwise uncomplicated cataract surgery. The nature of this rare condition remains unknown. Early anti-VEGF administration seems to demonstrate favorable results.

## Background

Cataract surgery is the most common operation performed in healthcare systems worldwide. Although relatively safe, intraocular infection following cataract surgery is a rare but dreaded complication that can have devastating consequences on vision. The incidence of endophthalmitis following cataract surgery is estimated between 0.04% and 0.27% [1–3]. To minimize this risk, prophylactic intracameral antibiotic use during cataract surgery has been proposed. In 2007, the European Society of Cataract and Refractive Surgeons (ESCRS) in a multinational, partially-masked placebo-controlled trial has provided strong evidence for using intracameral antibiotics in preventing postoperative endophthalmitis following cataract surgery [4]. In the wake of these results, endophthalmitis rates have dropped considerably in countries where intracameral injection was adopted as a routine method of prophylaxis at the close of cataract surgery. Therefore, in Europe, the use of intracameral antibiotics at the conclusion of the surgery represents the common practice. However, there is still debate regarding which is the optimal antibiotic to use.

Vancomycin is a branched tricyclic glycosylated non-ribosomal peptide produced by the Actinobacteria species *Amycolatopsisorientalis*. It is a broad-spectrum antibiotic that covers nearly all staphylococcal and streptococcal species, which are frequently encountered in postoperative endophthalmitis after cataract surgery [5]. Currently, it is the most commonly used prophylactic intracameral antibiotic during cataract surgery procedure in the United States [6]. Several studies have investigated its safety profile. Yoeruek et al. studied the toxic effects of cefuroxime and vancomycin on human corneal endothelial cells and found them safe in clinically used concentrations [7]. Higher concentrations could cause irreversible cell death. A recent randomized controlled trial examined the effects of intracameral vancomycin and gentamicin on macular thickness as measured by ocular coherence tomography. They found no statistically significant increase in macular thickness in the group that received intracameral vancomycin and gentamicin [8].

However, recent reports have demonstrated the causative role of vancomycin in the pathogenesis of Hemorrhagic Occlusive Retinal Vasculitis and suggest avoiding it for chemoprophylaxis. Nicholson et al. reported severe bilateral ischemic retinal vasculitis after uncomplicated cataract surgery in 2 patients [9]. Witkin et al. presented a retrospective case series of 36 eyes with postoperative HORV development after cataract surgery in which intracameral vancomycin was used [10]. Balducci et al. recently provided strong evidence of the causative role of vancomycin in HORV [11].

This case report describes the early anti-VEGF treatment strategy in a case of unilateral hemorrhagic occlusive retinal disease after uneventful cataract surgery which to the best of our knowledge has never been reported before.

## Case presentation

A 51-year-old healthy female underwent uneventful cataract surgery in her right eye. During the procedure, viscoelastic and vancomycin (1.0 mg/0.2 mL) were injected into the anterior chamber. Visual acuity on the first postoperative day was 20/20 and no other postoperative complication was detected.

On the seventh postoperative day the patient complained of acute painful visual deterioration. Visual acuity was 20/32 in her right eye with minimal anterior chamber and vitreous reaction, not suggestive of endophthalmitis. She was referred to our clinic 2 days later (post-op 9 days) for further investigation. Ophthalmic examination revealed best corrected visual acuity of 20/32 and 20/25 in the right and left eye respectively. Intraocular pressure was 15 mmHg in both eyes, pupil reaction was normal. Slit lamp examination of the right eye revealed 1+ cell in the anterior chamber and 1+ cell in the anterior vitreous. Fundus examination of the right eye was notable for mild vitritis, vascular attenuation, and peripheral artery occlusion, as well as scattered intraretinal and perivascular hemorrhages.

**Fig. 1:**
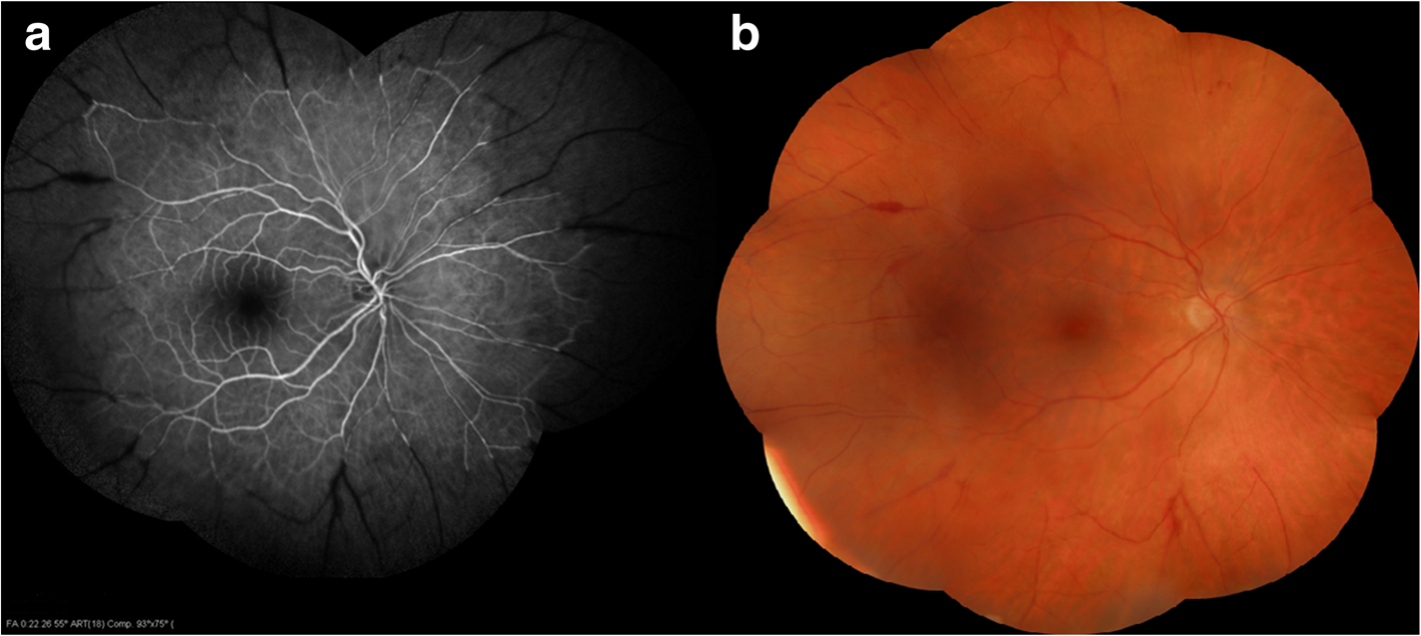
**a** Mosaic fluorescence angiography revealing peripheral vascular occlusion, extended areas of non perfusion, and scattered areas of perivascular staining **b** Mosaic color fundus photography showing vascular attenuation, peripheral artery occlusion and few scattered intraretinal and perivascular hemorrhages

**Fig. 2:**
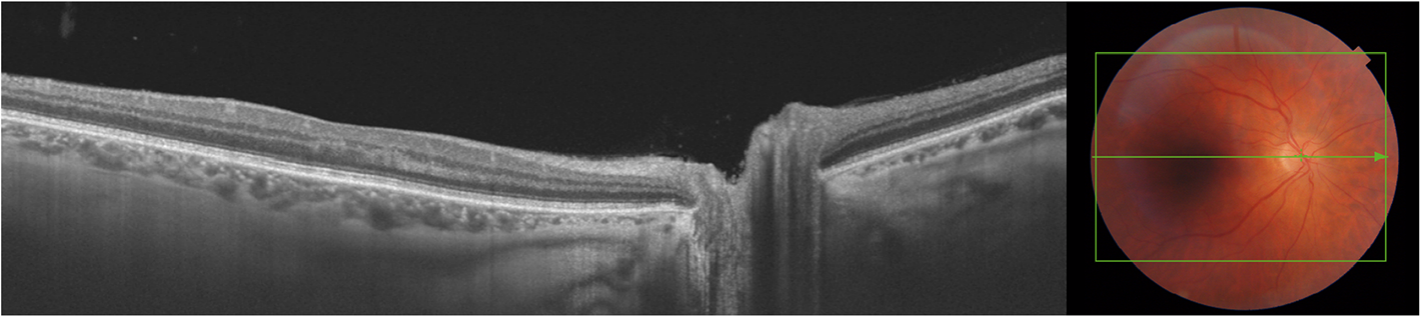
Macula Optical Coherence Tomography. Fovea architecture is intact with no sign of edema

Fluorescein angiography revealed delayed retinal filling, peripheral vascular occlusion, extended areas of non-perfusion, and scattered areas of perivascular staining (Fig. 1). Optical coherence tomography examination was unremarkable (Fig. 2). A 6 × 6 zone was acquired using optical coherence tomography angiography demonstrating the junction between perfusion and non-perfusion areas showing the choroidal vasculature intact.

Diagnosis of hemorrhagic occlusive retinal vasculitis was suspected based on the recent cataract surgery where intracameral vancomycin was used and characteristic clinical findings of the disease. As with previous reports of vancomycin-induced HORV, thorough systemic workup was unremarkable. Based on reports in the literature indicating the poor prognosis of the disease, the poor therapeutic effects of steroids and immunosuppressive treatment, we decided to proceed with early anti-VEGF treatment in an attempt to halt the progression of vision loss and reduce the risk of neovascular glaucoma. An intravitreal injection of bevacizumab (Avastin; Genetech, South San Francisco, CA) was administrated.

The following days the patient reported gradual pain relief and an improvement in her vision. Best corrected visual acuity was 20/25. Fundus examination showed no change, while fluorescein angiography showed reduced vascular staining. Οn the 25^th^post-operative day best corrected visual acuity was 20/20.

Despite visual acuity restoration, extended peripheral zones of non-perfusion were noted on fluorescein angiography. We discussed our concerns with the patient and we proposed continuation of anti-VEGF for at least 3 months to minimize the risk of neovascularization development. A total of three intravitreal injections were administrated in a 3-month period after surgery. We decided to proceed with photocoagulation treatment only in the occurrence of neovascularization.

At a 6-month follow-up examination best corrected visual acuity was 20/20 with no sign of neovascularization. Fundus examination and fluorescein angiography revealed no sign of disease activity. We decided to closely monitor the patient at regular intervals over the next 2 years in order to promptly, detect and treat a possible occurrence of neovascularization. The patient was provided a written informed consent in accordance with the tenets of the Declaration of Helsinki to having their medical data used for research purposes. Written informed consent was obtained from the patient for publication of this case report and any accompanying images.

## Discussion

HORV after uneventful cataract surgery is a dreaded complication. There is an ongoing debate concerning the association of intracameral vancomycin and HORV. Nicholson et al. reported severe bilateral ischemic retinal vasculitis after uncomplicated cataract surgery in two patients. The authors associated the vasculopathy with the use of intracameral vancomycin at the close of the cataract procedure [9]. Witkin et al. discussed the findings of 36 eyes that experienced severe HORV and found that this exceedingly rare condition could represent a delayed immune reaction to intracameral vancomycin [10]. Balducci et al. presented a case where HORV developed only on the eye where vancomycin prophylaxis had been used at the end of the procedure, while the second eye of the same patient that underwent cataract surgery without vancomycin prophylaxis remained unaffected [11]. Most reported cases present poor results.

The case presented herein showed some differences from other similar reports. First, our patient complained of painful progressive loss of vision. Ophthalmodynia was not described in other cases making the diagnostic procedure difficult as endophthalmitis could not be ruled out easily. The patient described the symptom as a dull, constant ache in the affected eye, which was relieved after anti-VEGF injection. We postulate that pain may be ischemic in origin similar to “ocular angina” presented in ocular ischemic syndrome [12]. Furthermore, fundus examination revealed few hemorrhages in contrast to the severe hemorrhagic component of previously reported cases. Moreover, OCT findings did not indicate macula pathology even though increased macula thickness and cystoid macula spaces have been described in other cases [10]. The absence of macular edema and photoreceptor disruption might explain the favorable final vision outcome.

Previous reports have presented several therapeutic approaches [9–11]. Despite intervention with high-dose topical and systemic corticosteroids, antiviral medication and early vitrectomy in many patients, visual outcomes were typically poor. Neovascular glaucoma developed in most patients and visual outcomes varied between no light perception to 20/50 [10].

In our case, we proceeded with early anti-VEGF treatment in our patient. Early outcomes were encouraging since our patient described an improvement in her symptoms. Three Intravitreal injections were performed monthly in order to halt disease progression and prevent neovascularization. At a 6-month follow-up examination the patient had 20/20 and no sign of vascularization.

This case report highlights the favorable outcome of immediate anti-VEGF therapy in cases of HORV. However, we have to take into account that the disease severity may vary and that in the presented case, HORV might have manifested with milder symptoms and a moderate visual acuity loss. Indeed, Lenci et al. reported a similar case with rapid resolution when treated with a short course of topical medications. The authors stated that the case probably represented the mild end of a spectrum of vancomycin toxicity [13]. Nevertheless, most reported cases had poor visual outcomes and further complications, thus we believe that in our patient early intervention might have modulated the course of the disease.

## Conclusions

To recapitulate, HORV is a devastating condition after otherwise uneventful cataract surgery. In our case, the early intervention with anti-VEGF treatment had a favorable result in restoring vision and preventing neovascularisation. Although it is difficult to draw accurate conclusions from single cases early anti-VEGF seems to have a positive role in stopping the cascade of HORV and preventing neovascularisation which if untreated would adversely affect patients vision and quality of life. Further studies are needed to ensure such a therapeutic effect.

## Abbreviations

HORV: Hemorrhagic Occlusive Retinal Vasculitis
OCT: Optical coherence tomography
VEGF: Vascular Endothelial Growth Factor
